# The Influence of Life Experiences on the Development of Resilience in Older People With Co-morbid Health Problems

**DOI:** 10.3389/fmed.2020.502314

**Published:** 2020-09-22

**Authors:** Gill Windle, Kate M. Bennett, Catherine MacLeod, The CFAS WALES research team

**Affiliations:** ^1^Dementia Services Development Centre Wales Research Centre, School of Health Sciences, Bangor University, Bangor, United Kingdom; ^2^School of Psychology, University of Liverpool, Liverpool, United Kingdom

**Keywords:** resilience, health, life course, adverse events, co-morbidites, healthy ageing

## Abstract

**Background:** Co-morbidity is a major late-life challenge with poor outcomes, yet many older people are resilient. We consider an ecopsychosocial framework of resilience to investigate this disparity. This theorises that sources of resilience may be personal, social and structural. We explored older people's responses and reactions to significant life experiences, to understand resilience development for managing later life health challenges.

**Methods:** We applied a two-stage, cross-sectional mixed-methods design to the Cognitive Function and Ageing Studies Wales (CFAS Wales). Participants' defined quantitatively as resilient (high level of well-being despite co-morbidity) were identified in the wave 1 dataset. A sub-sample of the resilient participants aged 65+ were randomly selected for semi-structured interviews (*N* = 20). Qualitative thematic analyses were both inductive and deductive.

**Results:** The analyses revealed four primary life experiences reflecting different developmental trajectories. “Early years as formative” and “work and employment as formative” occurred at normative developmental stages in the life-course. In contrast non-normative life events such as loss, bereavement, illness of self, and others underpinned the themes of “adverse events and experiences” and “caring experiences.” Four potential mechanisms for resilience were central to these life experiences, reflecting reactions, actions, and development: “character and self-identity;” “approach to life and insight;” “meaningful relationships and belonging.”

**Conclusions:** This work contributes further theoretical insights into the ecopsychosocial resilience framework. It highlights the process of interdependence between the individual and the wider environment, suggesting how the availability and accessibility of resources and human agency (protective factors), can influence, and be influenced by, the timing of significant events and experiences. In doing so, it corroborates international healthy ageing policy which recognises resilience as important for a public health response to support older people to adjust to changes and losses experienced in later life. It highlights the importance of current and future policies and services for supporting the management of adverse events earlier in the life-course, and recommends that policies and services take a “long view” on population health and well-being and consider the whole life-course, in addition to specific points in the ageing process.

## Introduction

In developed nations there has been an “epidemiologic transition,” reflecting the decline of infectious and acute diseases and a growth in chronic and degenerative diseases and co-morbidity (1). Comorbidity is associated with mortality, reduced quality of life and functional status, and increased use of health care (2–4). Older adults with increasing numbers of chronic conditions experience declines in life satisfaction (5). Declines in life satisfaction are associated with the prevalence of fair/poor general health and increases in disability, physical distress, mental distress, activity limitation, depressive symptoms, anxiety symptoms, sleep insufficiency, and pain (6).

Yet many older people with health problems do not necessarily see chronic conditions as an adversity or as a barrier to a good older age. These discrepancies between actual and perceived health are indicative of resilience in older age [e.g., (7)]. “Resilience” explains how some people have a good, or better than expected, outcome in the face of significant challenges. However, definitions of resilience vary. In response to the requirement for concept clarification a review and concept analysis of over 270 resilience research papers across the lifespan, synthesised with stakeholder perspectives, developed a working definition of resilience as “*the process of effectively negotiating, adapting to, or managing significant sources of stress or trauma. Assets and resources within the individual, their life and environment facilitate this capacity for adaptation and ‘bouncing back' in the face of adversity. Across the life-course, the experience of resilience will vary*” (8). This recognises that resilience is more than just an aspect of personality and has multiple sources.

International healthy ageing policy recognises resilience as important for a public health response that supports older people to navigate and adapt to changes and losses experienced in later life (9, 10). Despite this international interest, reviews conclude that far less is known about resilience in later life compared to childhood resilience [e.g., (11, 12)]. Quantitative research demonstrates the importance of resilience for well-being when living with ill-health (13), reducing the impact of a new chronic condition on disability (14), adjustment to multiple chronic conditions (15), and moderating the impact of daily stress on negative emotions (16). However, this does not reveal *how* resilience may have developed as these models are constrained by prior theoretical assumptions or limited data and, therefore, are unable to discover new processes (17). Several qualitative studies have explored resilience in older age [e.g., (18–20)], but, these studies have not focussed specifically on samples with ill-health. Moreover, little is known about the extent to which determinants of resilience earlier in the life course may influence later life challenges, especially when faced with a common adversity, particularly ill-health (12).

Longitudinal research on resilient children indicates that despite being born and raised in “high risk” circumstances (e.g., poverty, significant difficulties in their home environment) one third of these children went on to be competent, confident and caring adults (21). Some early life experiences might have contributed to success later, including opportunities to utilise diverse sources of support. Seery et al. (22) also found that the experience of some lifetime adversity predicted better mental health and well-being outcomes. This suggests that an adverse experience early in life rather than have a negative, sensitizing effect (perhaps due to pervasive deprivation and adversity), could have a strengthening “steeling” effect in relation to the response to adversity or stress in later life (23). Cumulative Inequality Theory (24) provides some explanation, recognising that personal trajectories are shaped by the accumulation of risk, available resources, perceived trajectories and human agency (2009, p. 334). The availability and accessibility of resources and human agency (protective factors), can influence, and be influenced by, the timing of significant events and experiences. Thus, the life course perspective represents a formal theoretical context within which the development of resilience can be embedded, as it reflects lifelong as well as age-specific challenges and resource opportunities.

Recognising that older age is shaped by prior experiences (25), we address the gaps in knowledge about resilience in later life. We apply a two-stage mixed methods design, first identifying a sub-sample of older people defined quantitatively as resilient (high level of well-being despite comorbidity) in order to explore the life experiences that may have enabled this resilience. We recognize that identifying and measuring resilience is a source of debate. A number of resilience measurement scales exist, although most measure factors that facilitate a resilient outcome, focusing mainly on psychological aspects (12). Contemporary research acknowledges that a quantitative evaluation of resilience should take into account two common components of resilience; the adversity, and the outcome of interest often referred to as “positive adaptation” (26). A key requirement is the adversity could ordinarily increase the risk of a poor outcome. The adversity could be biological, psychological, economic, or social (8). For example Joling et al. (27) operationalised dementia caregiver resilience as high levels of psychological well-being despite different types of high caregiving demands. Hildon et al. (28) operationalised resilience in older age as better-than-average quality of life in the face of significant adversity, such as ill-health, stress, changing life circumstances, being worse off financially, and experiencing a negative or difficult event such as bereavement.

**Figure 1:**
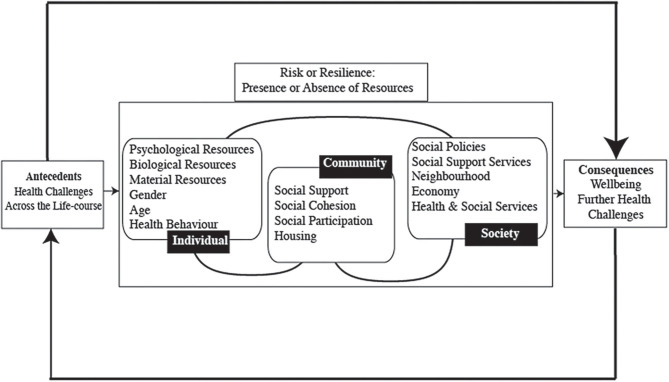
Resilience framework.

As previously outlined, comorbidity is associated with a number of poor outcomes, and is particularly pertinent given it is increasing likely to occur as people age (9). Hence comorbidity is the adversity chosen for this study. The second stage then qualitatively explores the participants' responses to significant life experiences as the context for the development of resilience. We draw on an ecopsychosocial resilience framework to understand experiences [Figure 1, adapted from (29)]. This recognises that resilience arises from the relationship between the individual and the environment around them, supporting people to deal with the health and social disadvantages they potentially face.

## Methods

### Participants

Data and participants are from the Cognitive Function and Ageing Studies Wales (CFAS Wales). This is a population-based study investigating change in health, well-being and cognitive function in community dwelling older people (including a proportion in care facilities) aged 65 years and over living in urban and rural areas of Wales. Participants were randomly sampled from general practice lists between 2011 and 2013, with equal numbers drawn from the age groups 65–74 and 75 and above (*N* = 3,593). Participants were interviewed in their own homes by a trained researcher. The response rate was 46%. Ethical approval was granted by North Wales Research Ethics Committee West. Participants first took part in main CFAS Wales interviews during 2011–2013, administered using assisted computer direct data entry.

This paper applies a cross-sectional mixed-methods design in two stages. First, we identified participants defined as resilient in the CFAS Wales wave 1 dataset. A systematic review of methodological approaches for measuring resilience found that “positive adaptation” had mainly been operationalised as the absence of psychiatric distress (e.g., no depression; no anxiety) in the face of an adversity, and no studies specifically measured positive mental adaptation and well-being, an important outcome in older age (26). Following the conclusions of Cosco et al., we operationalised resilience in this first step as a high level of well-being (a good outcome, as indicated by high life satisfaction) despite a significant adversity (two or more health problems). This identified potential participants, and a sub-sample were then selected for interview from those defined as resilient.

### Measures

High well-being was assessed with the Satisfaction with Life Scale (30). Five questions ascertain global cognitive judgments of satisfaction with one's life. Individual responses range from “1” (strongly disagree) to “7” (strongly agree). The final scale ranges between 1 (low satisfaction/extremely dissatisfied) and 35 (highly satisfied). Scores of 25 or more indicate high well-being.

Health problems were defined from the methodology of Brayne et al. (31). We identified participants who had experienced two or more of the following conditions: stroke, heart attack, angina, high blood pressure, diabetes, vascular disease, asthma, chronic bronchitis, arthritis, sight difficulties, and hearing difficulties that interfere with daily living, TIA, Parkinson's Disease, or epilepsy.

Participants were required to be cognitively capable of a discussion and were included in the sampling frame if their mini-mental status score (MMSE) >21. Scores range from 0 to 30, with higher scores indicative of better cognitive functioning (32). Demographic data were collected on age (years), gender (male/female), care setting (living in a care home/not living in care home), marital status (a dichotomous variable representing whether or not participants were married/cohabiting), education (years).

### Interview

A topic guide contained questions to encourage the participant to think about key events over their life. It was designed to capture unique experiences without explicitly referring to the theme of resilience in the interview. To mitigate against elements of deception around the nature of the interview, at the outset the interviewer clarified with the participant why they had been selected for this study. An early draft was discussed with the research team, refined and piloted (see Supplementary File 1). The semi-structured interviews took place during 2012–2013, in the homes of the participants at a time of their convenience. Participants understood they did not have to disclose details of past trauma and distressing experiences unless they wished to. The interviews were in English or Welsh, depending on preference. All interviews were audio-recorded. The semi-structured interviews were professionally transcribed, and then anonymised by a member of the CFAS Wales research team. Welsh interviews were transcribed in the original language and subsequently professionally translated into English. CFAS Wales study had a detailed data management plan and this was adhered to at all times.

### Data Analysis

We used a two-stage hybrid method for our qualitative analysis. First, we used the approach of IPA (Interpretive Phenomenological Analysis) to examine personal lived experience in detail, inductively interpreting how participants made sense and navigated their significant life experiences, rather than examining experience through a lens prescribed by pre-existing theoretical preconceptions (33). Each member of the team coded in detail the same two interviews, to establish a common view and to ensure agreement. The authors discussed the analysis on several occasions. Once all the interviews had been analysed individually, a thematic approach identified common themes, and further refinement made through discussions between all team members. We also independently read six interviews and produced summaries which were then compared with fully analysed interviews and discussed by the team. Finally, we synthesised the themes to identify significant life experiences and resilience resources.

## Results

Thirty-eight people were identified using random sampling, 20 interviews, lasting ~1 h, were undertaken. Participants' average age was 77 years (*SD* = 7 years), 12 were female, 10 were married, six widowed, two divorced, and two had remarried following a divorce. The average life satisfaction score was 29.15 (*SD* = 2.39), higher than the mean of the total sample (*M* = 26.84, *SD* = 5.23). The average number of health problems was 4 (*SD* = 2.0). More specifically, the number of conditions reported across the participants were angina = 4, diabetes = 9, Parkinson's Disease = 1, stroke = 3, epilepsy = 2, chronic bronchitis = 3, asthma = 2, arthritis = 14, hearing difficulties = 9, eyesight difficulties = 6, high blood pressure = 8.

There was a negative correlation between higher reports of health problems and lower life satisfaction for the total sample *r* (3,387) = −0.19, *p* = 0.001. This remained significant for those with two or more health problems *r* (2,222) = −0.16, *p* = 0.001, but not for those with one or no health problems *r* (1,165) = −0.04, *p* = 0.12.

**Figure 2:**
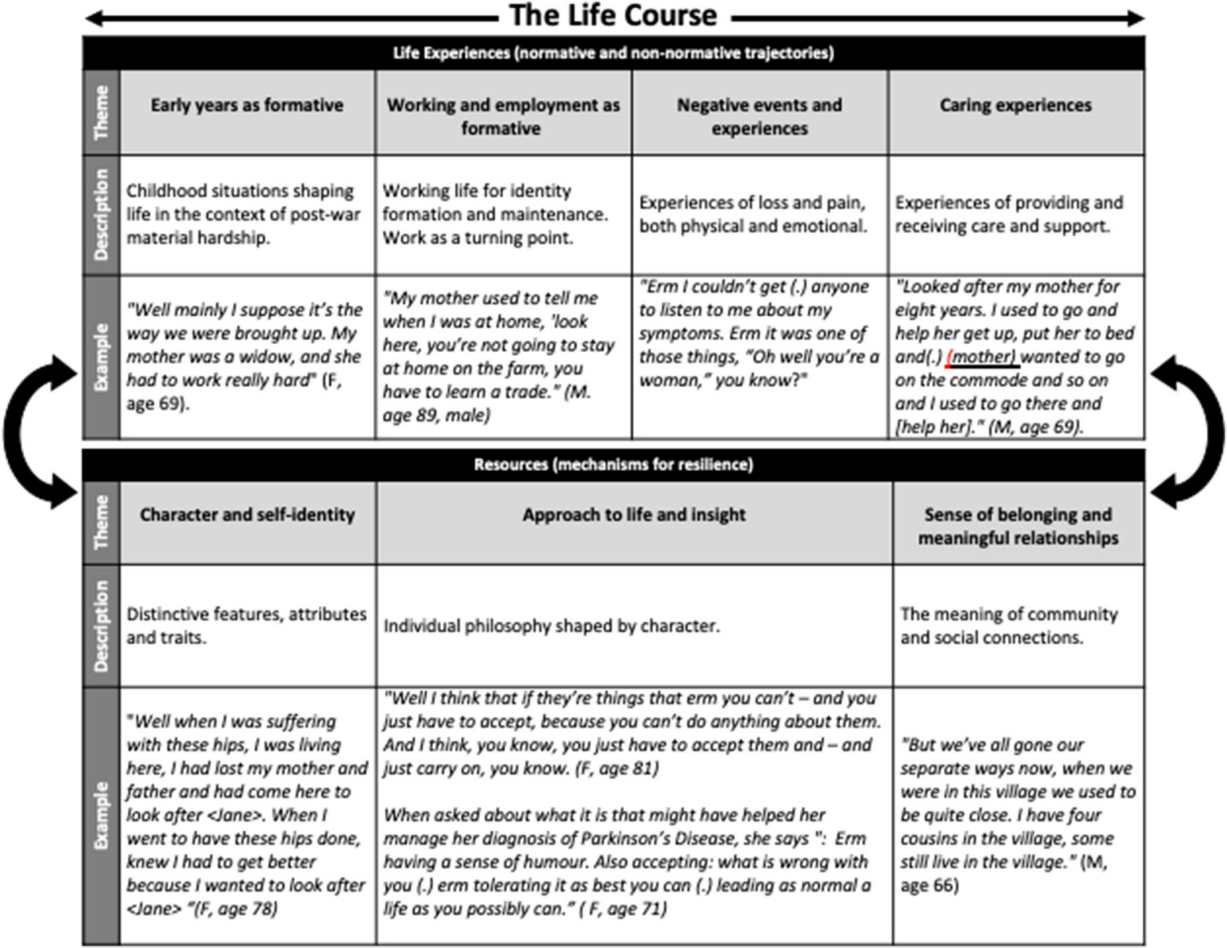
Life course experiences and mechanisms for resilience.

The analysis suggested four events representing both normative and non-normative experiences, (i.e., they occurred within or outside an expected developmental trajectory). These are*: “early years as formative;” “work and employment as formative;” “adverse events and experiences;”* and “*caring experiences*.” Central to these experiences were the participant's accounts of their reactions and subsequent actions, how they understood and managed the experience, which we define as: “*character and self-identity;” “approach to life and insight*;” and “*sense of belonging and meaningful relationships.”* These themes reflect an interplay between their development and application in the context of the life experiences, which can be described as resources, or mechanisms for resilience (see Figure 2). We illustrate each theme with examples, but the experiences and mechanisms for resilience are often inter-dependent and the interpretation synthesises these two elements to make sense of the narratives.

### Life Experiences

#### Early Years as Formative: “I'll Start With My Childhood, Because I Think That Explains a Lot”

Participants described childhood situations which shaped their subsequent lives, often in the context of material hardship from World War II and the early post-war years. The childhood perception of a family member making sacrifices was common. These experiences had a strong impact on identity development and strategies for dealing with subsequent challenges. When reflecting on her life and how she had managed her mother's manic depression, her father's Alzheimer's disease, her divorce and her own health problems (diabetes, arthritis, high blood pressure and eyesight difficulties affecting daily living), one participant remarked:

“*I look back on the past and think, “Well how did—what did mum and dad do?”… I just feel that you just have to take whatever comes and hits you. And, just get on with it really, because there are other people relying on you…. you just [(sighs)] you know, I've always been erm (.) what is it? Half—you know, is it half—half-full. (F, age 69)*.

This illustrates the importance of role models for learning and adaptation. Early experiences of helping their parents was important for several people and facilitated independence and a sense of family responsibility:

*My brother, he was 2 years younger than me, we were both young boys, and we use to go and help. My father worked on the farm next to where we lived, and he use to go around with a milk cart in those days, horse. ….. He use to get up at six o clock in the morning to deliver the milk and we helped him …. In a way it taught us a great deal, it was training, to prepare us for work*. *(M, age 89*).

School experiences were strong influences. Some participants described school as a happy time, but others described learning to tolerate difficulties in school, an important characteristic for managing future challenges. For Welsh speaking children, communication in school was particularly challenging, when many subjects were taught in English.

*I learnt the things, but I didn't know how to pronounce the words properly* (F, age 75).

Despite this, she achieved in subjects not requiring the use of English, reflecting an early capacity to negotiate a complex situation for the self as a source of resilience. She indicates how these early experiences and negotiations have facilitated her subsequent approach to life despite her ill-health (diabetes, asthma, arthritis and high blood pressure):

“*Nothing has left its mark on me for long*.”

Participants' early environments, although challenging, provided experiences for learning and the development of character, a resilience mechanism.

#### Working and Employment as Formative: “I Wanted to Do Something With My Life”

This theme occurred across all of the lifespan and was an important influence on identity formation and maintenance. For most, entering work was a turning point, influencing their future. One participant, who described herself as not “*very good at school*,” went on to further training and then ran her own small shop from late teens to retirement. Work was important in defining her character and approach to life. When discussing how she managed the loss of her husband and her own ill-health and disability (diabetes, arthritis, hearing difficulties affecting daily living), she remarked:

“*I suppose it's because I've always had to stand on my own two feet you know? Like having a business from an early age … you just do have to stand on your own two feet and get round everything and get on with it. You know, there is nobody to fall back on.”* (F, age 72).

Often work was a source of pride, pleasure and enjoyment, and later on for keeping busy and maintaining continuity. One participant, still farming in older age despite his health problems (stroke and arthritis), captures this, saying:

“*I think it would be quite dangerous to stop (.) I'm sure I'd be quite lost if I moved to a bungalow with no problems. Because I'm used to problems at work all the… Something goes wrong here every day, there's always something to do and paperwork—I'd miss that” (M, age 89)*.

Some participants were unable to enter their chosen profession demonstrated their ability to compromise and accept their situation.

“*Well I wanted to be a nurse, but mum was so poorly so there was more than enough work for me at home”* (F, age 84).

Others describe long hours in apprenticeships, factories or farms, yet needing to be valued “*I haven't come to do cheap labour, I've got to get on*,” (M, age 85). This early determination, to maximise a situation, along with compromise and acceptance reflect the development a strong sense of self and character, all important for managing sources of stress in later life. This participant reflects on the influence of his father's death on his young life:

“*I was young- determined to do something with my life—perhaps my life would have been completely different had my father been alive.”*

The determination fostered in early life continues into later life and how he managed his diabetes (he also reported eyesight and hearing difficulties affecting daily living):

“*but I was determined to know how to do it, perhaps I'm not a doctor or a nurse but I've learnt about diabetes. It hasn't stopped me, I went to hospital for a week at the beginning and I had to learn how to inject myself.”*

There are other examples where learning from experiences in earlier years has enabled participants to manage later challenges. For example, this participant found out early in life that keeping busy was helpful throughout her life:

“*Having plenty of work to do so you have to think of other people not yourself”* (F, age 75).

Another participant spoke at length about how her earlier experiences of being self-employed helped her become the woman she now was.

#### Adverse Events and Experiences: “I Don't See It as an Illness, I See It as a Challenge”

Experiences of loss and pain, both physical and emotional, resonate in the narratives. “*Things haven't been hunky-dory all the time, there have been problems.”* (M, age 70). Non-normative family bereavements occurred in many participants' childhoods and multiple losses were extremely painful. Determination resonates. This participant, living with arthritis and the effects of a stroke, who experienced the loss of four boys from his family, remarked:

“*Well I don't know, as one says,.. make the best of a bad situation, when you're determined”* (M, age 89).

This highlights how adaptive strategies that draw on personal resolve influence the experience's meaning and interpretation.

The significance of an early major event resonates with another participant, whose mother died of Tubercolosis, and who also contracted the disease herself and was hospitalised as a child for 2 years. She describes her emotional response to the loss:

“*When me mum died I thought, “Right, nothing worse than this can happen.” And then you get into yourself and think, you know, “It's my time now,” and get on with it.”* (F, age 74).

There is a realisation that this was the worst thing that could happen to her, and “life goes on” negating the impact of subsequent major events.

Many participants described the limitations brought about by their illnesses which affected them primarily in their older age. In the face of disabling arthritis, one participant remarks:

“*I want to walk and that's it, or try to walk. You've got pain after but I know what it is. I know what the pain is, it's arthritis and that, and there's nothing you can do about it.” So why should I waste my life worrying about that?”* (F, age 74).

One participant, who had been told in early adulthood she would be unable to have any more children, and who had significant mobility issues through arthritis in older age remarked:

“*Well I think the thing is you just have to accept things. And if you can't accept things, you're no good to anyone are you?”* (F, age 81).

Being determined, yet able to accept, are aspects of character which have developed and prevailed across the life course. Although there are experiences of loss and pain, character development is prominent. Those early experiences are important in positively re-framing illness in later life.

Participants discussed the ups and downs of their life history. For example, this participant reports she did not cope well with a diagnosis because it impacted on her work, which found rewarding:

“*Well I didn't cope with it in the beginning because I was working*, <*I worked*> *with the Social Services. And erm, that was what started off as Homecare obviously, and I did over 30 years service with that. Erm, and I really enjoyed my work.”* (F age 69).

She reported feeling envious of friend whose health problem was resolvable whilst hers was not. These emotions hindered, for a while, her resilience:

“*And a friend of mine now, she just had her hip done, and me being a bit jealous about that, because she's alright now. I mean I go into hospital and I'll come out exactly the same way practically.”*

This final example, demonstrates how on occasion, one event contributes, in this case the death of her mother and her own hospitalization, both to the dynamic nature of resilience:

“*No I never used to worry about anything; no I'd have enough worries when I was 10, so it doesn't bother me anymore. Nothing bothers—nothing bothers—when you lose somebody like that you're not worried about anything.”* (F age 74).

#### Caring Experiences: “It Didn't Feel Like Work, You See, It Was the Natural Thing to Do”

This theme reflects the experiences of providing care and support to others, but also receiving care and support from families, friends and professional services. This was often initiated in childhood, with participants caring for a sick parent.

“*And you know there was no electric lights, nothing was in them days, so I used to go down with me candle. She wanted a drink of tea which I had to make and her cream crackers. And I was thinking, “I don't mind doing this because I'll have one as well.” [(Laughs)] I always remember that. Nothing was too much to do at that age.” (F, age 74)*.

Although a child, she highlights a positive interpretation of an adverse situation, suggesting it provided something of value to the self. When discussing later life in the face of health and mobility restrictions she demonstrates this ability to re-interpret an adverse situation, “*I look forward to getting up every morning and I look forward to going to me bed at night, read me papers, watch me telly and that's it, that's my happiness.”* Developing the ability to identify value in less than desirable situations is an important aspect of resilience.

Giving care also enabled the focus to move away from participants' own problems. In early adulthood, in the face of her own grief at her mother's death another participant describes how she thought her father needed more help “*and in doing so you know, I dealt with the situation”* (F, age 71). This sense of purpose was a way of coping with her own loss. Family support is reciprocated across the generations, with older parents supporting their younger family members when experiencing earlier difficulties and can be interpreted as family resilience. This participant illustrates this from young adulthood:

I got married when I was twenty four, I had a daughter when I was 25, then I had two other children close to each other, and then my brother died when my children were quite young.…. I was 9 years older than him, so I used to look after him a lot I suppose. (F, age 75)

And later:

(“[Nephew]'s grandmother brought him up, his father suffered a stroke the same time, so [Nephew] used to come over to us. So that's why we've done so much with [Nephew], he didn't have any other family, so we've helped poor [Nephew]”

Throughout she describes a long history of family caregiving, but only towards the end of the interview does she express regret that she did not become a nurse.

### Resources (Mechanisms for Resilience)

#### Character and Self-Identity: “That's How It Was, We Were Determined”

Participants described their reactions to experiences and how they felt they dealt with them, particularly when faced with significant challenges. Tolerance and acceptance were common, suggesting that well-being was maintained in the face of challenges, particularly in later life, drawing on these characteristics. This woman remarks, when describing her hard working life in her teenage years, “*you got on with it and that was it, that's life*” (F, age 78). Later, when talking about her rheumatoid arthritis and osteoporosis she says:

“*I didn't ask for it to come, you can't take it away so I've just got to adapt to it. I can't do things that I would like to do, I just can't do it but I'm not sitting worrying about it. They tell me when I first was diagnosed with it they told me to walk about as walking gets oxygen into you bones. Well I've walked but it's not happening like it should and I can't do anything about it so it's there and I just have to live with it.”*

This draws attention to the continuity of aspects of character such as tolerance and acceptance despite challenges.

Determination, as already noted, is explicitly referenced across life-course experiences. This is illustrated by this participant: “*I dunno now, as I say, it just always been drilled into me, you just gotta do it and get on with it. … just determination.” (F, age 72)*. As she retired, she also lost her husband, yet notes “*I don't know, I suppose I just got on again really (.) just pick yourself up and carry on?”*

Despite their own difficulties, comparisons with others enables participants to provide a different perspective “*Always somebody worse off. But I remember saying that to the consultant and he said, ‘Yes, but it doesn't help you, does it?' I said, ‘It helps my mind.”' (F, age 69, with chronic bronchitis, arthritis and hearing difficulties affecting daily living)*. This attribute enables a re-appraisal of the situation, again underpinning continuity. There is an intrinsic need to either maintain or restore equilibrium, to preserve the self.

Participants displayed several characteristics which appeared to facilitate successful psychological adjustment. This draws attention to a recognition of knowing that life will contain events that could be considered less than desirable, and a need to draw on the self to turn this into something that can be managed and avoid or reduce the impact.

#### Approach to Life “You Have to Look on the Bright Side of Life, That's What I Think”

The characteristics inferred from the participants' narratives were important in understanding how they dealt with their concerns and reactions to events over the life-course. This approach to life involved their understanding their situations, and actively managing them. Focusing on the present—“*just not think about anything—just live for today and not think about tomorrow”* (F, age 74)—reflects an important perspective for managing the self when faced with very difficult ill-health. This also reflects a temporal aspect, a focus on the present-time in later life, which is further articulated “*you have to deal with things, if you don't, then you make life miserable for the children, don't you. And this and the other, you have to cope with things as they happen* (F, age 75). Not worrying too much and having a positive outlook featured as an important perspective when faced with challenges “*Hard work doesn't kill anyone. It's worry that kills people.”* (M, age 85).

Keeping active and occupied was seen as important. In discussing his brother in law's death 1 year after retiring, one participant describes his understanding of this early death:

“*He stopped working you see, he stopped doing what he used to do, it's unwise for anyone who's used to working to stop like that. It's better if you look for something to do, it doesn't matter what, even if you do voluntarily without any pay”* (M, age 89 living with arthritis and the effects of a stroke).

The awareness that change at this transition point potentially represents a detrimental break in continuity is important for strategies to keep mentally and physically active. The role of activity and occupation, despite ill-health was also important for remaining socially engaged “*well if you stay at home, you'll only get depressed”* (F, age 75, living with diabetes, asthma and arthritis). Religion was important for many participants both for staying socially connected and providing meaning for life events. In discussing his mother's death, one participant notes “*Other people came to offer their sympathy and so on but there was something that was part of me that helped me. I can't explain, it was my belief that helped me most.”* (M, age 66, living with diabetes, epilepsy, chronic bronchitis, and hearing difficulties affecting daily living

An ability to “keep going” was observed by one participant in early life, who noted:

“*My mother developed arthritis quite badly soon after (Participant's brother died) and she couldn't move. That shook us all but then you have to carry on when you have three children, you just had to, there wasn't any choice.”*

This earlier observation was reflected when talking about her own health difficulties (she was living with diabetes, arthritis, asthma, high blood pressure, and also reported Polymyalgia Rheumatica):

“*I'm better than I've been for a while, I can move better, much better than I used to. One of my friends for example, she doesn't see anyone all day long where she lives, so she's [decided] to move to a home. I don't understand [her], I'll keep going until I can't cope any more, for example, if I couldn't move, but while I can move I'm staying here, I'm staying put. [(laugh)]”*

#### Meaningful Relationships and Belonging “'Cause He Looked After Us, We Looked After Him”

Participants discussed their connections with friends and family, set in the contexts of the broader importance of attachments to the home and the locality for feelings of cohesion. For all participants, despite experiences of loss and hardship, family attachments are important: relationships between parents, siblings, children and spouses are an important resource for support. In describing the family's response to the early death of her young nephew, one participant captures this, saying “*well I don't know, I don't know how we got through it. We supported each other”* (F, age 78). Participants described family as important in times of crises. Family responsibility, having family members to look after, experiences of having been looked after as a child all motivated ongoing reciprocal exchanges of support.

Broader friendships in the community were also an important resource for emotional support and sharing problems “*as you get older, it's good to have someone your own age that you can talk things over with”* (F, age 78). Others had friends and neighbours who they felt would provide practical support if they needed it, as one participant notes,

“*I got the bug 1 day and I didn't go out, and they noticed that my car hadn't moved from here, and they phoned to ask, ‘are you alright?' ‘yes'. So they would help you, I fell and hurt myself once, and [Friend] came over to ask ‘what do you want?”'* (F, age 75).

As with family relationships, reciprocity was part of friendships, facilitating ongoing exchanges. The same participant describes how her neighbours call on her for help.

“*They turned to me the other day, one of the girls was ill and (neighbour) came here, can you come over, isn't well.”*

However, “*there isn't much of a community now.”* (M, age 70) reflects the changing nature of local life that resonated in older age.

A sense of belonging through connections to communities, meaningful relationships within and between friends and family within these communities and beyond, provides an important resource for support at times of need, but also through providing support to others, enables reciprocal exchanges to continue to feel valued and needed, facilitating individuals to be resilient in the face of chronic health conditions.

## Discussion

We explored how life experiences influenced the development of resilience, by interrogating narratives of significant events or stressful experiences in the life-course and how these were managed, to understand the meaning of those experiences. The study focuses on the experiences of resilient older adults, as indicated by high life satisfaction despite facing adversity (in this study co-morbid health conditions). The results yield important insights from a life-course perspective to understand resilience. Fundamentally, we suggest that later life resilience is determined by both normative and non-normative experiences that influence the development of resources, which we term “resilience mechanisms.” Two of these mechanisms can be seen as individual factors of the ecopsychosocial model: character and self-identity; and approaches to life and insight. The other two mechanisms reflect both community and societal resources: a sense of belonging and meaningful relations. In turn, these mechanisms potentially determine the impact of subsequent challenges, illustrating resilience as a dynamic process, and contesting perspectives which argue that resilience is inherent in the “self.” Collectively our findings provide support for the ecopsychosocial resilience framework, as participants drew on personal, social and structural aspects, highlighting the interrelationships between the individual and their wider environment.

Elsewhere, gerontologists interested in resilience in later life share similar thinking around the conceptualisation of resilience, contributing to the advancement of theory. For example, Wiles et al. (19) qualitatively explore resilience in older people in New Zealand. They found that internal, or personal characteristics of resilience such as positive attitude or purpose in life were deeply embedded in social and environmental contexts that could underpin or undermine resilience. They argue that “*resilience should be seen as a contextualised process which can be both individual and environmental*” (p. 416). Wild et al. (34) examined the utility of the concept of resilience for critical gerontology. They suggested some key principles for its use, notably that individual resilience should be situated within other levels of resilience such as neighbourhood, community and society, recognising the interdependence between the different levels of older people's lives. In Canada, Wister et al. (35) reviewed and synthesised the literature to suggest a life-course model of multi-morbidity resilience which represents a set of risk or protective traits, resources and processes that occur over the life-course to promote resilience. Taken together, gerontologists are developing the evidence base for resilience in later life to be defined as a process of interdependence between the individual and the wider environment where aspects of the environment can enable the personal strengths to adapt and manage. This complements resilience research undertaken with children and adolescents [e.g., (36–38)].

Although all our participants experienced adverse events, none disclosed any information about serious maltreatment in childhood. This does not necessarily mean this did not happen to the participants, but we allowed people to tell us what was important to them and did not explicitly ask about serious adverse childhood experiences. This is an important consideration, as significant, persistent maltreatment in childhood may lower the ability to be resilient compared to life events [see (23), for discussion]. However, positive, normative experiences in later life, such as marriage, employment, and family relationships (as noted in our study) are suggested as “turning points” by others, countering the effects of significant early adversities such as maltreatment [e.g., (39)]. Nevertheless, it would appear from our exploration that the participants' experiences of life events were to some extent time-limited adverse experiences, such as childhood bereavement. Whilst they did not prevail over long periods of time, the long-term consequences are known to be detrimental to the mental health of adults (40). Still, rather than letting those experiences influence their lives adversely, they utilised those experiences to build their characters, allowing them to reframe their later life illnesses positively. The early deprivation experienced by some participants did not prevail across the life span, as participants drew on, and developed, their characters to take advantage of employment opportunities, for example.

Similarly, participants drew strength from their caring experiences to foster later life resilience. Not only did those experiences develop character, sense of purpose and skills but they also encouraged connectedness and reciprocity with family, friends, and neighbours, which persisted into later life, either concerning long-standing relationships but also developing new ones. These experiences may have enabled the strengthening or “steeling” effects suggested by Rutter (23), and provide further support for the conclusion of Seery et al. that, “*in moderation, whatever does not kill us may indeed make us stronger”* [(22), p. 1,025]. More broadly, considering this in the context of Cumulative Inequality Theory (24) the availability of resources and human agency, described here as resilience mechanisms, may have modified any potential negative outcome posed by the risks from adversity, such as health difficulties.

### Implications for Policy and Practice

This dynamic nature of resilience as highlighted in our research is acknowledged by the World Health Organisation as a target for public health policy and important for Healthy Ageing (9). As more longitudinal studies of resilience emerge which consider the life-course perspective, the challenge will be for policy makers and practitioners who currently focus on discrete areas of development (e.g., geriatrics, pediatrics) to take the “long view” on population health and well-being and work together to develop a life-course approach to support healthy development.

Of course, it will always be important to focus on specific phases of development. Therefore, not only should a long view forward be taken by policy makers but also a long view backward, when working with older people. This long view backward could consider not only individual experiences but also the historical and cultural context which formed the experiences and characters of older people. By drawing on successful past personal and community experiences of overcoming challenge, one may be able to provide older people with the tools, often already existent, to face and overcome challenges of ageing.

The World Health Organisation's European Strategy for Child and Adolescent Health (41) recommends adopting a life-course approach to support development, acknowledging that health, and illness in adulthood are embedded in health and experiences in previous stages of the life-course. In our study, “early years as formative” and “work employment as formative” had a strong impact on the participants' development. The findings suggest the environment gives opportunities for the individual's learning and the development of the distinctive features, attributes and traits of their character. This work can begin in childhood, as noted by others, and can continue throughout adulthood [e.g., (21–23)]. Also important is providing opportunities for people to reflect on their own development and in times of stress draw on both their personal strengths and on the strengths in their communities. As the ecopsychosocial resilience framework suggests, it is the collective and collaborative impact of these factors which promote resilience.

A suggestion from this study could be that utilising this framework in both policy and in practice within a life-course perspective will enable societies to both support those resilient people to remain resilient but also to identify areas for action to facilitate resilience in those who are not yet resilient or who are vulnerable. These policies and practices need to be implemented at the individual, community and societal levels. Our data suggests specific areas of policy. For example, enhancing social support from families and communities, fostering social cohesion, and strong neighbourhoods could promote resilience (42). Where informal social support is not available, polices are required to ensure that more formal or statutory social support is in place.

Other aspects of intervention might focus on the individual, primarily drawing on psychological therapies. Indeed, there is a growing global interest in resilience-building interventions, as captured in systematic reviews. Macedo et al. (43) concluded that despite evidence to suggest that resilience promotion interventions were effective, the interpretations were hindered by the poor methodological quality of available studies. Moreover, no interventions were targeted at older people, and all were psychological interventions. Whilst Leppin et al. (44) included some patients with chronic conditions in their review, many of the included studies used a wide array of conceptualisations in relation to intervention application. There was some evidence to suggest the interventions were effective, but the authors indicate the studies were poorly specified and lacked theoretical clarity. None specifically targeted older people. Another systematic review of randomised controlled trials (RCTs) or controlled trials assessing the efficacy of programmes designed to develop, enhance or improve resilience in adults showed that interventions using mindfulness or CBT techniques appeared to enhance measures of individual resilience. Unfortunately, no descriptions of the study samples are included, so it is not possible to ascertain if any of these programmes included, or specifically targeted, older people (45). Despite the potential, resilience interventions have yet to be developed and tested with older people specifically and have not yet translated into models of service delivery for older people.

### Strengths and Limitations

The mixed methods approach to resilience is unique. To date most research on resilience has either taken a quantitative approach or a qualitative approach. In this study we have identified, quantitatively, a sample of older people who have high levels of life satisfaction despite experiencing health comorbidities, following the approaches of others for operationalising resilience in cohort data [e.g., (26–28)]. These participants can be defined as resilient. This approach was only possible because this study is embedded within a large-scale cohort study.

Quantitative research has shown resilience is important for maintaining well-being in the face of chronic illness (13), reducing the impact of a chronic condition on disability (14) and adjustment to multiple chronic conditions (15). Our research adds qualitative insights into how older people may develop the resilience measured quantitatively in these previous studies. Qualitative research can provide context and can contribute new theoretical understandings to the study of resilience in later life (17), and the meaning of experiences (23). Thus, some important factors may be missing or lacking in sufficient detail in quantitative studies to understand the nuances which underpin resilience and its mechanisms. The current study goes some way to address those deficits. In turn, it provides further insights for future quantitative models to test, thereby making a major contribution to resilience research.

We thought carefully about the design of the qualitative aspects, using a transparent and consistent approach to research design and conduct, derived from a framework for excellence in qualitative research (46). Notable in our research was the attention to detail in the process of coding and interpreting themes, involving considerable discussion between the authors of the meaning and interpretation of the participant's narratives. Participants' were not explicitly asked about resilience. Interpreting how they made sense and navigated their significant life experiences was the challenge of the analysis.

There are two limitations in our design. First, it is retrospective, and one could argue that our participants are looking back through “rose-tinted glasses.” However, there are two counter arguments to that. By using a life-course perspective in a time-unlimited interview we are giving participants the opportunity to reflect on their lives, and our experience elsewhere suggests that positive response sets are less likely in those situations (47). In addition, our focus is on resilience in later life, and therefore, one should not dismiss the positive self-reflections on past life, because that may indeed be a mechanism for later life resilience. Second, our study focuses on those who are resilient, and we do not have a non-resilient comparison group. Thus, we are not able to say for certain whether our themes and mechanisms would be present for those non-resilient participants. Other work has found differences between resilient and non-resilient participants at all levels of the ecopsychosocial framework (18, 48) and further work could usefully explore the extent to which our findings replicate in other groups of older people, and identify the unique aspects that enable resilience. An optimal research design could follow people over the whole life course to see how life experiences help develop resilience, and how in turn resilience is useful for managing chronic conditions. Unfortunately this was not possible for this study. However, future research could expand on this work to look at the life-course events of both resilient and non-resilient participants with chronic conditions to see how these may differ. The focus of our study was the development of resilience and we chose co-morbid heath difficulties as the contextual adversity. Future studies could select different adversities pertinent to older age (e.g., the onset of cognitive impairment) or consider a different approach in the data collection such as more explicit questioning around illness experiences.

We are also unable to say whether their hardships were objectively normative or non-normative. Given the age of our cohort, many people who were born at this time would have experienced similar levels of hardship in the war and post-war years. One might argue that our participants had experienced enough stability to provide the foundations for resilience. To address this question, we would need to examine the life-course of non-resilient participants.

## Conclusion

Our work is important for its contribution to four areas: method; theory; knowledge; and application. The study has a distinctive mixed-methods approach with a strong sampling approach. Uniquely, we drew our qualitative sample from a large panel dataset, using classical sampling methods. Our sample comprised older people who had high levels of life satisfaction despite facing significant health challenges. In addition, the interview was novel in two ways. First, the interview followed a life course perspective, whilst second, avoiding explicit questions about resilience. Thus, our findings reflect non-primed responses. The study provides an important contribution to the theory of resilience and the development of resilience. It demonstrates the importance of resources at individual, community, and societal levels to the development of resilience across the lifespan. Our findings highlight the importance of both normative life events such as early years and work, and non-normative events such as adverse life experiences (e.g., bereavements), and caring experiences, on the development of resilience for older people. The results also provide insight into the mechanisms which facilitate resilience. Collectively, these findings demonstrate how the availability and access to the resources interact with the timing of significant life events and experiences to facilitate (and one could argue, hinder) resilience. Finally, the implications of our work provide some early indication that it is not only important to take a forward view when thinking about service provision and ageing well, but also a long backward view. Understanding the life experiences of older people is essential if we are to provide appropriately tailored services and resources for older people that they consider appropriate and are willing to use.

## Data Availability Statement

The datasets generated for this study are available on request to the corresponding author.

## Ethics Statement

The studies involving human participants were reviewed and approved by North Wales Research Ethics Committee West. The patients/participants provided their written informed consent to participate in this study.

## Author Contributions

GW and KB were CFAS Wales co-investigators. GW designed the qualitative sub-study, developed the topic guide, oversaw the data collection, and led the writing of the manuscript. KB contributed to the writing, editing of the early drafts, and manuscript. CM contributed to quantitative data collection, oversaw the qualitative data management, and contributed to the writing of the final manuscript. All authors developed the coding system, undertook data analysis, and interpretation.

## Conflict of Interest

The authors declare that the research was conducted in the absence of any commercial or financial relationships that could be construed as a potential conflict of interest.
