# Supplementary material for: The Influence of Life Experiences on the Development of Resilience in Older People With Co-morbid Health Problems
Source: Front Med (Lausanne). 2020 Sep 22;7:502314. doi: 10.3389/fmed.2020.502314 (PMC7536341; doi:10.3389/fmed.2020.502314)
Supplement: Supplementary file 1 [file Table_1.docx]

**INTERVIEWER TO READ**

***Introduction to the participant***

“I’d like to talk to you today about how you deal with things, and what has made you who you are. You might not realize this, but we can see from our data that although you have reported some health problems, these do not seem to have any major adverse effects on your well-being. Other people in a similar situation to yourself do not have the same outcome, and I’m really interested in talking to you about this. This will mean me asking you some questions about your life history, this is your story. There are no right or wrong answers, this is more like a one sided conversation, where I might say very little.”

**EMPHASISE – make clear to the participant that they do not have to disclose/describe details of any past trauma.**

**___________________________________________________________________**

**Questions**

- Looking back on your earlier years (childhood, adolescence, early adulthood), can you think of any examples of things that might have been stressors/major events/sources of worry that had a strong impact on you?
- Can you remember how you felt at that time?
- What is the most important thing that has happened to you in your life?

**Instruction to interviewer:**

*Explore through prompts* ***–*** *the meaning and impact of* *early experiences, some of the non-normative events; changes in family, friends, schools, services; personality/characteristics; transitions to adolescence and early adulthood – turning points; goals over the life course – any changes made?*

*____________________________________________________________________*

**Questions**

- In general, when you have been in a stressful situation or under pressure [as per examples], what aspects of your life help you or what do you do to get through it?
- Who/what can you really count on when things aren’t going well? How do you go about this?

**Instruction to interviewer:**

*Explore - descriptions of the strategies and resources that lead to managing these situations/risks/events – prompts from resilience framework – e.g. the role of personal characteristics, family, neighbourhood, services, etc. examples of times when you had to really exceed yourself.*

**Questions**

- Thinking about your life story, what would you say are your strengths and qualities?
- How do you think you have acquired these, and how do they help you to be strong in life?
- Do you feel like you’ve successfully made it through any problems? If so, what helped you make it through? If not, what prevented you?

**Instruction to interviewer:**

*Explore the present time – the meaning from the earlier events/stresses and resources used.*

To end:

- Besides the things you’ve just talked about, is there anything else you would like to add about other things that have made you the person you are today?
